# COVID-19 and Breast Cancer in Brazil

**DOI:** 10.3389/ijph.2023.1605485

**Published:** 2023-03-03

**Authors:** Aline Ferreira Bandeira Melo Rocha, Ruffo Freitas-Junior, Glalber Luiz Rocha Ferreira, Danielle Cristina Netto Rodrigues, Rosemar Macedo Sousa Rahal

**Affiliations:** ^1^ Postgraduate Program in Health Sciences, Federal University of Goiás, Goiânia, Brazil; ^2^ Advanced Center for Breast Diagnosis (CORA), Federal University of Goiás, Goiânia, Brazil; ^3^ Goiás State Education Department, Goiânia, Brazil

**Keywords:** breast cancer, COVID-19, Brazil, breast cancer screening, clinical staging of breast cancer

## Abstract

**Objectives:** This study aimed to evaluate COVID-19 effects on breast cancer screening and clinical stage at diagnosis in patients of 50–69 years of age receiving care within the public healthcare network (SUS) in 2013–2021 in Brazil and its macro-regions.

**Methods:** This ecological study used Poisson regression to analyze trends in screening and staging. A secondary database was formed using SUS sources: outpatient data system of the SUS network and Oncology—Brazil Panel.

**Results:** There was a reduction in screening, with an annual percent change of −5.9 (*p* < 0.022). The number of notified cases fell by 31.5% in 2020–2021 compared to 2018–2019. There was a 10.7% increase in the proportion of stage III/IV cases (*p* < 0.001) in 2020–2021 compared to 2013–2019, now surpassing the number of cases of early stage breast cancer.

**Conclusion:** COVID-19 led to a reduction in breast cancer screening and an expressive increase in advanced tumors in users of the public healthcare network. Urgent interventions in public policies are required as the negative effects of the pandemic on the diagnosis/treatment of breast cancer are becoming apparent even earlier than expected.

## Introduction

The COVID-19 pandemic impacted healthcare services worldwide due to the need to prioritize resources for urgencies and reduce dissemination of the virus at healthcare services (1). Consequently, there were negative repercussions on elective procedures, including cancer screening (2–4), which was paused in Brazil and in the vast majority of countries (2, 5, 6).

According to data published by the World Health Organization, over half the countries investigated interrupted treatment services for hypertension either partially or totally, while 49% interrupted the treatment of diabetes and its complications; 42% interrupted cancer treatment; and 31% suspended cardiovascular emergencies. Furthermore, 50% of these countries reported a reduction in public screening programs (7). National and international medical societies published recommendations on cancer care, taking individual risks and benefits to patients into consideration (4, 8–11).

As the pandemic progressed and vaccination programs were implemented, the restrictive measures hitherto imposed were eased somewhat, allowing healthcare managers and professionals to evaluate whether to begin offering elective procedures, particularly the screening, diagnosis and early treatment of malignant neoplasms (3, 12). Deciding on these issues was of utmost importance in view of the real risk that the delay in cancer treatment during the pandemic would increase cancer-related morbidity and mortality (13).

In addition to the set of restrictive measures implemented as public policies to combat the pandemic, many women opted to delay healthcare treatment because they were worried about COVID-19 infection and its complications (14).

In this respect, breast cancer was particularly concerning, as it is the most common form of cancer, with an estimated 2.3 million new cases diagnosed annually worldwide (15). Many medical associations voiced their concerns regarding the impact of the restrictive measures on breast cancer (9, 10, 16). Indeed, studies published in the literature have already shown a decrease in the number of breast cancer diagnoses and in breast cancer screening, with no sign of a return to pre-pandemic levels (17–20). However, the eventual repercussions of this difficulty in providing appropriate care were yet to be ascertained, particularly with respect to diagnosing cases at more advanced stages of the disease (18).

With the pandemic still ongoing and the general lack of data on the subject, the objective of the present study was to evaluate the possible decrease in breast cancer screening and the increase in the percentage of advanced clinical stage in women aged 50–69 years, compared with previous years, in Brazil. This age group constitutes the brazilian target population recommended in the Guidelines for the Early Detection of Breast Cancer (21).

## Methods

This was an ecological study designed to evaluate temporal changes in which data on breast cancer screening and on the clinical stages of breast cancer were analyzed for the period from 2013 to 2021 with respect to women of 50–69 years of age receiving care within the public healthcare system. Data were analyzed for Brazil as a whole and for its five geographical regions (the north, northeast, southeast, south and mid-west).

A secondary database was formed using various different sources: data on the number of mammograms performed were extracted from the outpatient data system of the SUS network (SIA/DATASUS) (22), while the data on clinical staging at diagnosis were obtained from the SUS’s Oncology—Brazil Panel (DATASUS) (23). In addition, the data used to calculate the target population in the country were acquired from the Brazilian Institute of Geography and Statistics (IBGE) (24), and the data used to calculate the size of the female population covered by supplementary healthcare were obtained from the National Agency for Supplementary Healthcare (ANS) (25).

The internal review board of the Teaching Hospital, Federal University of Goiás approved the study protocol under reference CAAE 56747022.0.0000.5078. Since the study was conducted using freely available, unrestricted secondary data, the requirement for signed informed consent was waived.

### Target Population

The intercensal projected population of Brazil established by the “Projections of the population of Brazil and Federation Units by sex and age: 2010–2060” (24), updated in 2018, was used for the 2013–2021 period. From this population projection, the percentage of women who had private health insurance was extracted for each year of the period studied (data available at ANS).

### Breast Cancer Screening Coverage

The number of exams carried out annually between 2013 and 2021 was obtained from the DATASUS outpatient database (22). Two procedure codes were taken into consideration: bilateral screening mammogram (02.04.03.018-8) and mammogram (02.04.03.003-0 40). As far as data on breast cancer screening are concerned, underreporting is unlikely, since healthcare institutes need to register the mammograms performed in order to receive payment for services provided.

Accordingly the Technical Parameters for Breast Cancer Screening (26), to provide a more realistic estimate of the number of procedures performed, the breast cancer screening coverage goal to be achieved at the established period took the supplementary healthcare coverage at each location into account so as not to overestimate the procedures available within the public healthcare program.

Therefore, the percentage of women with private health insurance were obtained from the ANS and was subtracted from the target population (25). Based on the information obtained, the expected number of mammograms for the year in question was calculated.

The expected number of exams for the target population was calculated from the total number of women of 50–69 years of age, the percentage of women with private health insurance was subtracted from the target population in accordance with the recommendations of the National Cancer Institute for two-yearly screening (26).

Estimated coverage was calculated based on biannual screening of 100% of the target population. This indicator was expressed as a percentage and calculated from the ratio of the number of mammograms performed and the number of expected mammograms for the target population (26, 27).

### Clinical Staging

In order to extract the data on clinical staging from the oncology panel platform (23), the only filter applied was that used to select breast cancer stages I, II, III and IV, referring to the stage registered at chemotherapy, radiotherapy or both. These data were grouped as follows: 1) stages I/II, representing the initial stages of breast cancer, and 2) stages III/IV, representing advanced stages of the disease. Stage 0, which refers to ductal carcinoma *in situ*, a pre-cancerous manifestation that is not considered breast cancer, was excluded.

### Statistical Analysis

Trends in the rates of breast cancer screening were evaluated according to the annual percent change (APC) in the estimated breast cancer screening coverage in Brazil and its regions. The Poisson regression model was applied in the analysis using JoinPoint Regression, version 4.9.0.1 (28). The 95% confidence intervals (95%CI) were calculated, with *p*-values <0.05 being considered statistically significant.

In the analysis and interpretation of the results, an increase in estimated breast screening coverage was assumed when the APC was positive, and the minimum value of the confidence interval was above zero. Conversely, breast-screening coverage was considered to have decreased when the APC was negative, and the maximum value of the confidence interval was below zero. Coverage was considered to have stabilized when, irrespective of the amount of coverage, the minimum value of the confidence interval was below zero and the maximum value was above zero.

Staging was characterized using absolute and relative frequencies calculated for Brazil as a whole and for the different regions. Comparison of staging prior to the pandemic (2013–2019) and during the pandemic (2020–2021), for Brazil and for the different regions, was performed using McNemar’s test. Poisson regression analysis was used to analyze trends in staging between 2013 and 2021. Coverage in the pre-pandemic period and coverage during the pandemic were compared using the Mann-Whitney U test. The data were analyzed using the Statistical Package for the Social Sciences (SPSS), version 26.0 (29). A 5% significance level was adopted (*p* < 0.05).

## Results

A total of 1,613,119 mammograms were performed within the public healthcare service in Brazil in 2020, a number that is 40% lower than the 2,658,289 mammograms performed in 2019. In 2021, this number was 2,189,734, still 18% lower compared to 2019.

**FIGURE 1:**
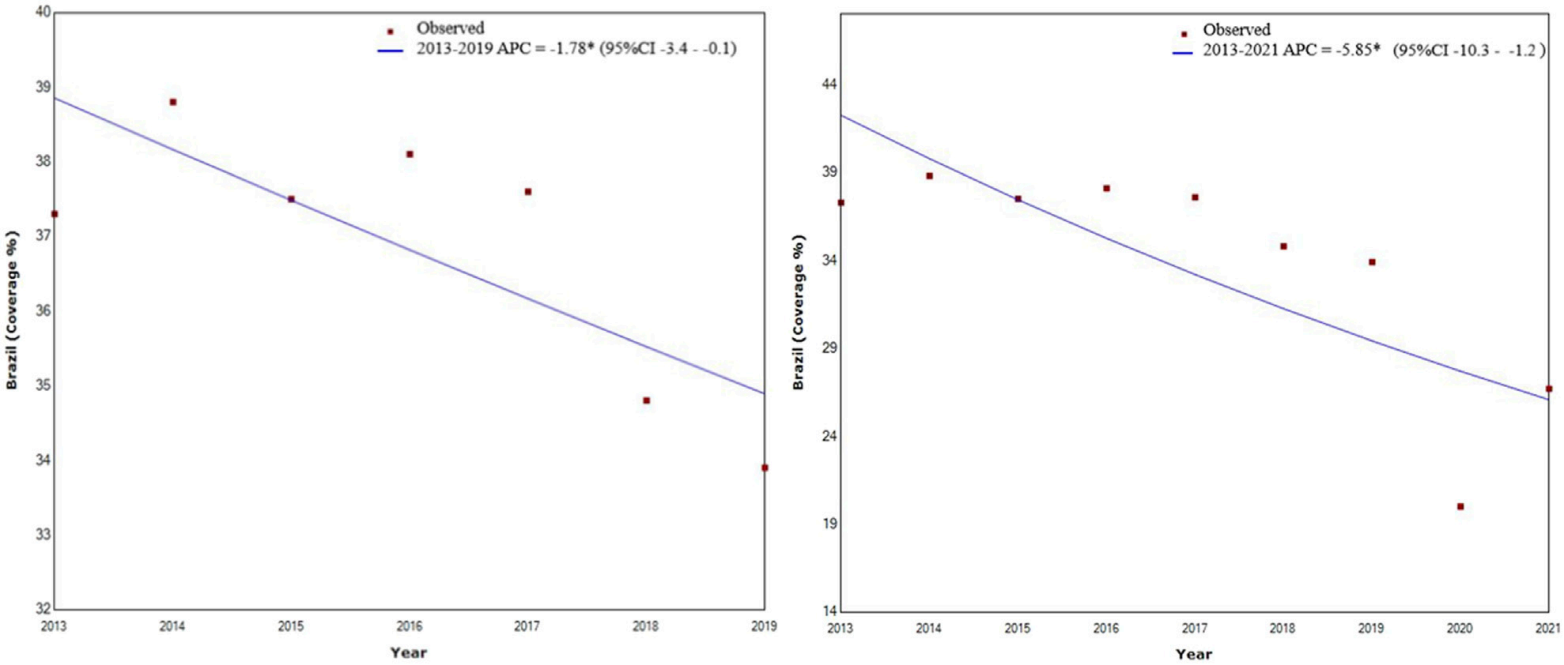
Trend in the rate of breast cancer screening coverage within the public healthcare system in Brazil between 2013–2019 and 2013–2021 in women of 50–69 years of age (Poisson regression model). Brazil, 2013–2021. APC, Annual Percentual Change; 95%CI, 95% Confidence Interval.

A reduction in breast cancer screening coverage was found for the 2013–2019 period, with an APC of −1.78 (*p* < 0.040; 95% CI −3.4–−2.8), while for the 2013–2021 period, was found an APC of −5.85 (*p* < 0.02; 95% CI −10.3–−1.2), as shown in Figure 1. Compared to 2019, coverage decreased by 41% in 2020 and by 21% in 2021 (Table 1).

**TABLE 1:** Percentage rate of breast cancer screening coverage in Brazil as a whole and in its individual regions in women of 50–69 years of age receiving care within the Brazilian public healthcare service (SUS) between 2013 and 2021 (Brazil, 2013–2021).

Coverage/Region	Year								
	2013	2014	2015	2016	2017	2018	2019	2020	2021
Brazil	37.3	38.8	37.5	38.1	37.6	34.8	33.9	20.0	26.7
Region									
Midwest	20.6	20.4	15.4	17.1	16.0	15.4	18.0	9.6	15.3
North	15.0	18.0	15.7	13.4	15.2	12.9	12.7	9.9	11.2
Northeast	27.7	29.1	29.4	32.0	32.4	26.4	25.6	14.1	21.8
Southeast	47.4	51.3	49.5	48.6	47.1	45.5	43.8	26.7	34.0
South	44.9	42.4	41.7	42.9	42.4	41.5	40.2	23.5	29.8

When the 2013–2019 and 2020–2021 periods were compared, a reduction was found in the mean rate of breast cancer screening coverage in Brazil from 36.71 ± 1.73 to 21.97 ± 3.02 (*p* < 0.01). A similar reduction was found in all the regions of the country: mid-west: from 17.50 ± 2.01 to 11.15 ± 2.52 (*p* < 0.001); northeast: from 28.79 ± 2.50 to 16.30 ± 3.49 (*p* < 0.001); north: from 14.55 ± 1.70 to 10.24 ± 0.57 (*p* < 0.001), southeast: from 47.41 ± 2.35 to 28.85 ± 3.28 (*p* < 0.001), and south: from 42.24 ± 1.39 to 25.29 ± 2.87 (*p* < 0.001), as shown in Table 2.

**TABLE 2:** Comparison of breast cancer screening coverage between 2013–2019 and 2020–2021 (Brazil, 2013–2021).

Coverage/Region	Period (years)		*p*-value*
	2013–2019	2020–2021	
Brazil	36.71 ± 1.73	21.97 ± 3.02	**<0.001**
Region			
Midwest	17.50 ± 2.01	11.15 ± 2.52	**<0.001**
Northeast	28.79 ± 2.50	16.30 ± 3.49	**<0.001**
North	14.55 ± 1.70	10.24 ± 0.57	**<0.001**
Southeast	47.41 ± 2.35	28.85 ± 3.28	**<0.001**
South	42.24 ± 1.39	25.29 ± 2.87	**<0.001**

Data are expressed as means ± standard deviation. * Mann-Whitney U test.

Bold was used to demonstrate the statistical significance of the *p* value.

During the 2018–2019 period, a total of 26,675 cases of breast cancer were registered for the country as a whole compared to 18,284 in 2020–2021, representing a reduction of 31.5% and highlighting the impact of the pandemic on the total number of cases identified.

In parallel, when 2013–2019 was compared to 2020–2021, a 10.7% increase was found in the proportion of cases of stage III/IV breast cancer (*p* < 0.001), which now surpass the proportion of cases of stage I/II for Brazil as a whole. Analysis of the different regions showed a statistically significant proportional increase in advanced stages, with a 21.3% increase in the mid-west, a 10.3% increase in the northeast, 16.5% in the north, 11.2% in the southeast and 6.2% in the south (Table 3). In all the regions except for the south, the percentage of cases of stage III/IV surpassed that of stage I/II during the pandemic (Table 3).

**TABLE 3:** Breast cancer stages at diagnosis prior to the COVID-19 pandemic (2013–2019) compared to those during the pandemic (2020–2021) for Brazil as a whole and its regions (Brazil, 2013–2021).

Stage/Region	Period (Year)		Total	*p*-value*
	2013–2019	2020–2021		
Brazil				
I and II	48,567 (59.2)	8,865 (48.5)	57,432 (57.3)	**0.001**
III and IV	33,440 (40.8)	9,419 (51.5)	42,859 (42.7)	
Midwest				
I and II	2,378 (54.2)	345 (32.9)	2,723 (50.1)	**0.001**
III and IV	2,008 (45.8)	704 (67.1)	2,712 (49.9)	
Northeast				
I and II	11,387 (56.9)	2,138 (46.6)	13,525 (55.0)	**0.001**
III and IV	8,631 (43.1)	2,454 (53.4)	11,085 (45.0)	
North				
I and II	1,310 (55.4)	254 (38.9)	1,564 (51.9)	**0.001**
III and IV	1,053 (44.6)	399 (61.1)	1,452 (48.1)	
Southeast				
I and II	22,426 (60.2)	3,940 (49.0)	26,366 (58.2)	**0.001**
III and IV	14,847 (39.8)	4,103 (51.0)	18,950 (41.8)	
South				
I and II	11,066 (61.6)	2,188 (55.4)	13,254 (60.5)	**0.001**
III and IV	6,901 (38.4)	1,759 (44.6)	8,660 (39.5)	

Data are expressed as absolute frequency (relative frequency). * McNemar’s test.

Bold was used to demonstrate the statistical significance of the *p* value.

**FIGURE 2:**
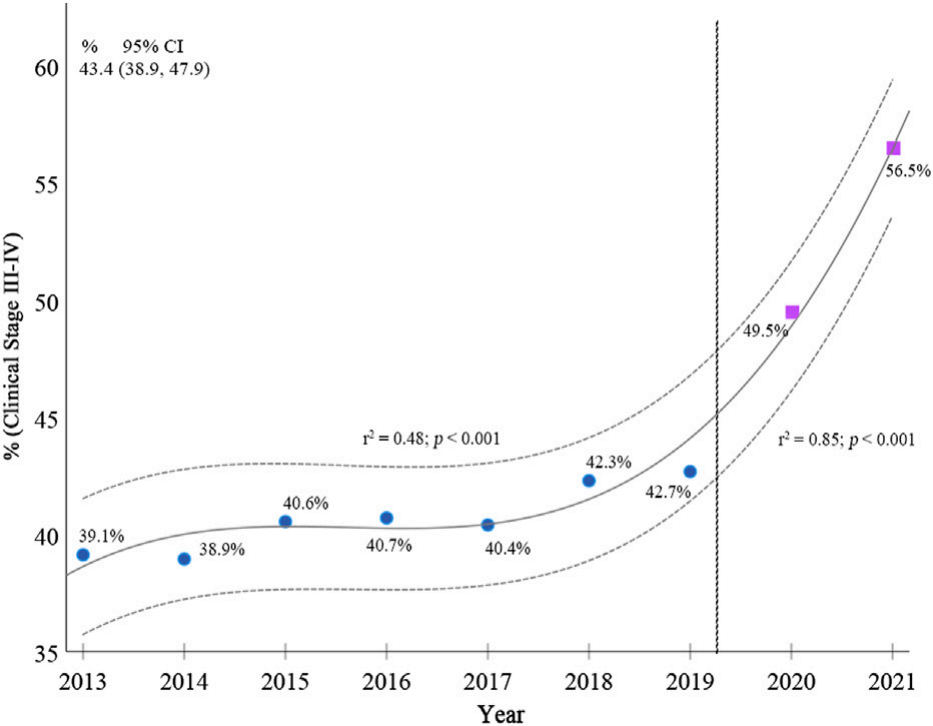
Breast cancer stages from 2013 to 2021 in women of 50–69 years of age (Poisson regression model). Brazil, 2013–2021.

According to the Poisson regression analysis, when comparing the coefficient of determination (r2) of the trend in clinical staging of breast cancer from 2013–2019 (r2 = 0.48; 95%CI 38.9–47.9; *p* < 0.001) with 2013–2021 (r2 = 0.85; 95% CI 38.9–47.9; *p* < 0.001), there is an increase in staging III and IV in the period that includes the pandemic over the percentage in 2013–2019, as shown in Figure 2.

## Discussion

The results of the present study show that during the pandemic breast cancer screening coverage in women in the target group established by the Ministry of Health decreased in Brazil as a whole and in all its regions. Even after the restrictive measures implemented in the country had eased, breast cancer screening coverage failed to return to pre-pandemic levels. This finding is in line with observations from several countries (18), with a possible impact on the diagnosis of breast cancer, its staging, treatment and associated mortality rates.

In Brazil, cancer screening and diagnosis were more severely affected than treatment, irrespective of the type of cancer (11). A reduction was found in the number of breast cancer diagnoses, with the cases detected suggestive of a poorer prognosis (30), as also shown in our study. The proportion of palpable nodules was significantly greater in 2020 compared to 2019 (31).

A reduction of 40% was found in breast cancer screening in Brazil between 2019 and 2020. Other studies have reported reductions ranging from 35.3% to 42% (11, 31). In 2021, even after the restrictive measures had been eased and vaccine programs implemented, breast cancer screening coverage remained 21% lower compared to the years that preceded the pandemic. This finding is concerning in a country in which breast cancer screening coverage was already poor, as shown in the present study and in previous publications (27, 32, 33).

The inequalities that exist in Brazil were compounded by the effect of the pandemic. As shown, there was a greater reduction in the screening rate in the regions of the country in which the human development index (HDI) is lower such as in the north, northeast and mid-west. This finding is in agreement with various studies conducted in Brazil showing the correlation between breast cancer screening and regional differences in the availability of healthcare services (32, 34, 35).

The present study showed a reduction in the absolute numbers of cases of cancer notified and a proportional increase in the number of cases diagnosed at more advanced stages during the COVID-19 pandemic, with the number of cases of stages III and IV now surpassing that of stages I and II in all the regions of the country except for the south. Studies conducted both in developed and in developing countries (36, 37) have also shown a reduction in the number of cases diagnosed (17, 37) and an increase in the number of diagnoses at more advanced stages (30, 38).

To the best of our knowledge, the present study is the first to evaluate data for Brazil as a whole and for its individual regions, with the results highlighting the pronounced consequences of the pandemic throughout the entire country for patients within the public healthcare network. Prior to the pandemic, 40.8% of cases of breast cancer in women of 50–69 years of age were diagnosed at stages III/IV, with this proportion increasing significantly to 51.5% between 2020 and 2021.

The increase in breast cancer diagnoses at advanced stages in Brazil as a consequence of delayed diagnosis could result in an increase in more mutilating surgeries and a need for adjuvant and neoadjuvant treatment that could have been avoided (1, 39). Other factors to take into consideration are a possible increase in the number of avoidable deaths (40), deterioration in the quality of life of surviving patients and a rise in health-related expenditure (1, 40).

The impact on morbidity and mortality will depend on the duration of the interruption to screening. For this reason, restoring trust and guaranteeing the safety of patients participating in screening programs is crucial (39, 40), as is the implementation of actions aimed at raising awareness and encouraging screening (19).

Interventions in the form of public policies need to be implemented urgently in an attempt to increase screening and patient access, expanding coverage beyond the levels seen prior to the pandemic. These actions are crucial in order to minimize the consequences of delayed diagnosis and treatment of breast cancer patients, bearing in mind that the negative effects of the pandemic are becoming apparent earlier than expected.

### Limitations

Since the data were obtained from a secondary source, the analysis was restricted to the data supplied from SIA/DATASUS, the Oncology-Brazil Panel, ANS and IBGE, and this constitutes a limitation of the present study. There is a possibility that cases of cancer registered with the Oncology-Brazil Panel could be underreported. About breast cancer screening, the data are quite reliable because mammograms are paid for only when they are recorded in the system. Therefore, the likelihood of underreporting is minimal.

### Conclusion

During the COVID-19 pandemic, there was a significant reduction in the number of mammograms performed within the public healthcare system in Brazil, with a reduction in breast cancer screening coverage together with an expressive increase in the number of patients with advanced tumors (clinical stages III and IV) at presentation. The speed at which this increase in advanced stages of the disease occurred is noteworthy and indicates that breast cancer screening should be reinitiated as quickly as possible in order to avoid the impact of late diagnosis.
